# Benefit of [18F]-FDG PET/CT for treatment-naïve nasopharyngeal carcinoma

**DOI:** 10.1007/s00259-021-05540-8

**Published:** 2021-09-01

**Authors:** Shan-Shan Yang, Yi-Shan Wu, Wei-Chao Chen, Jun Zhang, Su-Ming Xiao, Bao-Yu Zhang, Zhi-Qiao Liu, En-Ni Chen, Xu Zhang, Pu-Yun OuYang, Fang-Yun Xie

**Affiliations:** 1 Department of Radiation Oncology, Sun Yat-Sen University Cancer Center, State Key Laboratory of Oncology in South China, Collaborative Innovation Center for Cancer Medicine, Guangdong Key Laboratory of Nasopharyngeal Carcinoma Diagnosis and Therapy, No. 651 Dongfeng Road East, Guangzhou, 510060 China; 2 Department of Nasopharyngeal Carcinoma, Sun Yat-Sen University Cancer Center, State Key Laboratory of Oncology in South China, Collaborative Innovation Center for Cancer Medicine, Guangdong Key Laboratory of Nasopharyngeal Carcinoma Diagnosis and Therapy, No. 651 Dongfeng Road East, Guangzhou, 510060 China; 3 Department of Head and Neck, Sun Yat-Sen University Cancer Center, State Key Laboratory of Oncology in South China, Collaborative Innovation Center for Cancer Medicine, Guangdong Key Laboratory of Nasopharyngeal Carcinoma Diagnosis and Therapy, No. 651 Dongfeng Road East, Guangzhou, 510060 China; 4 Department of Nuclear Medicine, Sun Yat-Sen University Cancer Center, State Key Laboratory of Oncology in South China, Collaborative Innovation Center for Cancer Medicine, Guangdong Key Laboratory of Nasopharyngeal Carcinoma Diagnosis and Therapy, Guangzhou, China

**Keywords:** PET/CT, Nasopharyngeal carcinoma, Induction chemotherapy, MRI

## Abstract

**Background:**

To test the advantages of positron emission tomography and computed tomography (PET/CT) for diagnosing lymph nodes and staging nasopharyngeal carcinoma and to investigate its benefits for survival and treatment decisions.

**Methods:**

The performance of PET/CT and magnetic resonance imaging (MRI) in diagnosis was compared based on 460 biopsied lymph nodes. Using the propensity matching method, survival differences of T3N1M0 patients with (*n* = 1093) and without (*n* = 1377) PET/CT were compared in diverse manners. A radiologic score model was developed and tested in a subset of T3N1M0 patients.

**Results:**

PET/CT performed better than MRI with higher sensitivity, accuracy, and area under the receiver operating characteristic curve (96.7% vs. 88.5%, *p* < 0.001; 88.0% vs. 81.1%, *p* < 0.001; 0.863 vs. 0.796, *p* < 0.05) in diagnosing lymph nodes. Accordingly, MRI-staged T3N0-3M0 patients showed nondifferent survival rates, as they were the same T3N1M0 if staged by PET/CT. In addition, patients staged by PET/CT and MRI showed higher survival rates than those staged by MRI alone (*p* < 0.05), regardless of the Epstein-Barr virus DNA load. Interestingly, SUVmax-N, nodal necrosis, and extranodal extension were highly predictive of survival. The radiologic score model based on these factors performed well in risk stratification with a C-index of 0.72. Finally, induction chemotherapy showed an added benefit (*p* = 0.006) for the high-risk patients selected by the model but not for those without risk stratification (*p* = 0.78).

**Conclusion:**

PET/CT showed advantages in staging nasopharyngeal carcinoma due to a more accurate diagnosis of lymph nodes and this contributed to a survival benefit. PET/CT combined with MRI provided prognostic factors that could identify high-risk patients and guide individualized treatment.

**Supplementary Information:**

The online version contains supplementary material available at 10.1007/s00259-021-05540-8.

## Introduction

Nasopharyngeal carcinoma is a specific head and neck cancer with unique geographical and ethnic distribution. Approximately 133,354 new cases occurred in 2020, with the highest incidence in southern China [1]. Radiotherapy is the main treatment modality for early-stage nasopharyngeal carcinoma, while concurrent chemoradiotherapy with or without induction chemotherapy is recommended for locoregionally advanced nasopharyngeal carcinoma.

Conventional work-ups, including head and neck magnetic resonance imaging (MRI), chest X-ray or computed tomography, abdominal sonography or computed tomography, and bone scans, are recommended for tumor, node and metastasis (TNM) staging. For patients with bilateral enlarged lymph nodes or palpable lymph nodes below the cricoid cartilage, [18F]-Fluorodeoxyglucose (FDG) positron emission tomography and computed tomography (PET/CT) is highly recommended because of the high risk of occult distant metastasis [2, 3]. For N0-1 patients with Epstein-Barr virus (EBV) DNA less than 4000 copies/mL, a prior study [3] insisted on a low risk of distant metastasis and the comparable value of conventional work-up versus PET/CT for initial staging and finally did not recommend PET/CT due to its cost. Additionally, a recent study confirmed that there was no survival benefit of adding PET/CT to conventional work-up for stage I–II nasopharyngeal carcinoma [4]. We suppose that precise detection of metastatic cervical lymph nodes and correct N-stage perhaps more affects the prognosis of these patients, instead of focusing on the value of detecting occult distant metastasis. Stage T3 nasopharyngeal carcinoma without distant metastasis is the most typical representation. For example, patients with T3N0M0 can achieve comparable overall survival by intensity-modulated radiotherapy alone as patients with stage II disease [5], whereas the risk of distant metastasis is as high as 18% at 3 years after radical chemoradiotherapy for T3N2-3M0 nasopharyngeal carcinoma, and induction chemotherapy followed by concurrent chemoradiotherapy is strongly recommended for these patients [6–8]. Therefore, we aimed to determine whether PET/CT can influence the prognosis of nasopharyngeal carcinoma by providing an accurate diagnosis of metastatic lymph nodes.

Currently, the optimal treatment mode for the subgroup of T3N1M0 remains the most controversial [9, 10]. T3N1M0 patients have locoregionally advanced disease, but clinical trials that justified the benefit of induction chemotherapy [8] did not include this type of patient. A retrospective study found no survival benefit of additional induction chemotherapy for these patients [9], whereas male patients staged with T3N1M0 and EBV DNA higher than 2000 copies/mL were the target population, as suggested by another study [10]. Although EBV DNA showed prognostic value, it made little sense in clinical practice due to the lack of a unified test standard, robust serum level, and accepted cut-off value. Considering the generality of PET/CT and MRI in different hospitals, we aimed to identify an approach to individualized treatment by developing a radiologic score in a cohort of T3N1M0 nasopharyngeal carcinomas.

## Methods

### Patients and study design

Cohort A included 460 cervical lymph nodes from 336 patients who underwent node fine-needle aspiration biopsy guided by ultrasonography and PET/CT and MRI examinations before treatment to test the performance of PET/CT in diagnosing metastatic lymph nodes. Cohort B consisted of 1093 T3N1M0 nasopharyngeal carcinoma patients who received both PET/CT and head and neck MRI, while cohort C included 1377 T3N1M0 patients who underwent MRI alone. Cohort B and cohort C were compared to find the survival benefit of adding PET/CT to MRI. Specifically, 838 patients in cohort B who received concurrent chemoradiotherapy with or without induction chemotherapy were identified as cohort D to analyze the benefit of induction chemotherapy. The flowchart is presented in Supplementary Fig. 1. All patients were restaged based on the 8th edition American Joint Committee on Cancer/Union for International Cancer Control (AJCC/UICC) staging system. This study was approved by the Sun Yat-sen University Cancer Center Institutional Review Board (No. B2021-059–01).

### Imaging analysis

**Fig. 1:**
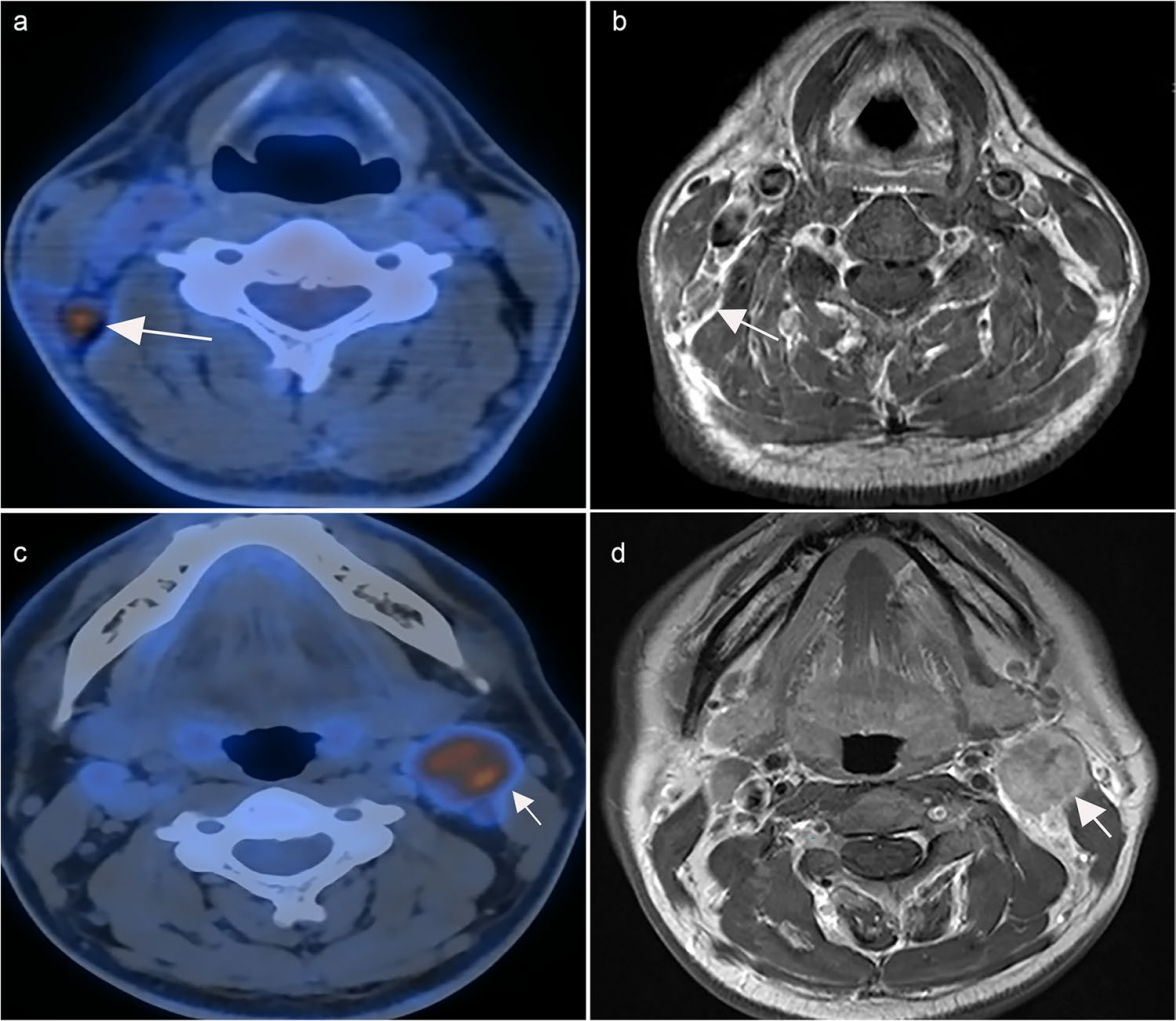
Cervical lymph nodes in PET/CT (left) and contrast-enhanced T1-weighted MRI (right), **a** PET/CT correctly diagnosed positive lymph nodes, while MRI diagnosed negative lymph nodes by mistake (**b**); both PET/CT (**c**) and MRI (**d**) correctly detected metastatic lymph nodes. Abbreviations: MRI, magnetic resonance imaging; PET/CT, [18F]-fluorodeoxyglucose positron emission tomography with computed tomography

The PET/CT and MRI protocols are described in the Supplementary Methods. MRI images were read by two experienced radiologists, and PET/CT was read by two experienced nuclear physicians who were blinded to the MRI results. Any differences were resolved by consensus. Metastatic lymph nodes were diagnosed according to the radiologic criteria [11]: (1) retropharyngeal lymph nodes with a minimal axial diameter of 5 mm or greater and cervical lymph nodes with a minimal axial diameter of 10 mm or greater; (2) minimal axial diameter of 8 mm for clusters of 3 or more lymph nodes; and (3) lymph nodes with necrosis or extranodal extension. Similar to a previous study [12], radiologic extranodal extension was categorized into 4 grades: grade 0, no extranodal extension; grade 1, invasion to surrounding fat; grade 2, coalescent nodes; grade 3, infiltrating adjacent structures. As reported [13], the diagnostic criteria for nodal necrosis based on MRI included (1) focal area of low signal intensity on T1-weighted images with or without enhanced edges and (2) focal area of high signal intensity on T2-weighted images. On PET/CT, lymph nodes were considered positive when [18F]-FDG uptake increased significantly compared with the background [14]. In the final decision-making, the PET/CT results were supplemented with the MRI findings (Fig. 1).

### Treatment and follow-up

All patients received intensity-modulated radiotherapy. The prescribed doses were 66–72 Gy to gross tumor and lymph nodes. The treatment modality included concurrent chemoradiotherapy with or without induction chemotherapy, radiotherapy alone, and induction chemotherapy plus radiotherapy. Induction chemotherapy regimens included docetaxel plus cisplatin and 5-fluorouracil, cisplatin plus 5-fluorouracil, docetaxel plus cisplatin, and gemcitabine plus cisplatin every 3 weeks for two to four cycles. For concurrent chemotherapy, weekly cisplatin or a 3-weekly cisplatin regimen was administered. After treatment, follow-up examinations were conducted at least every 3 months during the first 2 years and then every 6 to 12 months thereafter. Examinations including EBV DNA testing, nasopharyngoscopy, head and neck MRI, chest X-ray or computed tomography, and abdominal sonography or computed tomography were performed regularly. PET/CT was recommended if distant metastasis or locoregional recurrence was still uncertain by regular examination during the follow-up period. Recurrence or metastasis was confirmed by biopsy if possible.

### Statistical analysis

Failure-free survival (FFS, time from diagnosis to treatment failure or death) was defined as the primary endpoint, and the secondary endpoints were distant metastasis-free survival (DMFS, time from diagnosis to distant metastasis or death), locoregional relapse-free survival (LRRFS, time from diagnosis to locoregional recurrence or death), and overall survival (OS, time from diagnosis to death).

Categorical variables were compared by the chi-square test. The cut-off values of the continuous variables were determined by receiver operating characteristic curve (ROC) analysis. In cohort A, McNemar’s paired-sample test or chi-square test was used to compare PET/CT and MRI in terms of sensitivity, specificity, positive predictive value, and negative predictive value. In cohort B, Kaplan–Meier survival curves stratified by MRI-based N stage and PET/CT-based T stage were evaluated by the log-rank test. The propensity score matching (PSM) method was applied to balance confounders between cohort B and cohort C at a ratio of 1:1. To confirm the benefit of PET/CT in N0-1 patients with EBV DNA less than 4000 copies/mL, the cut-off value of EBV DNA was 4000 copies/mL in cohort B and cohort C [3]. The survival rates of MRI and PET/CT plus MRI were compared by the Kaplan–Meier method. Univariate and multivariate Cox regression analyses in the PSM cohort were performed to determine independent factors. In cohort D, the PSM method was also performed to balance confounders between patients receiving concurrent chemotherapy with or without induction chemotherapy at a ratio of 1:1. The cut-off value was 2000 copies/mL in cohort D to compare with a model reported in a previous study [10]. Univariate and multivariate Cox regression analyses in PSM cohort D were conducted to select independent factors for risk stratification. The survival curves of the patients receiving concurrent chemotherapy with or without induction chemotherapy were also compared. The model was evaluated by the concordance index (C-index) and compared with a previous model [10] by computing the *p* value using the “survcomp” package in R. Statistical analysis was conducted by R 4.0.1 (http://www.r-project.org/) and SPSS 26.0. A two-sided *p* < 0.05 was considered to be statistically significant.

## Results

### Advantage of PET/CT vs. MRI in diagnosing cervical lymph nodes

In cohort A, 58.0% of patients (195/336) were diagnosed with stage III, and patients staged N1 accounted for 77.1% (259/336) (Supplementary Table 1). Among the 460 biopsied cervical lymph nodes from 336 patients, 269 (58.5%) and 191 (41.5%) lymph nodes were pathologically positive and negative, respectively. Among them, 96.7% (260/269) of positive and 75.9% (145/191) of negative lymph nodes were correctly detected by PET/CT, while only 88.5% (238/269) of positive and 70.7% (135/191) of negative lymph nodes were correctly diagnosed by MRI. PET/CT was significantly more sensitive than MRI for detecting cervical lymph node metastasis (*p* < 0.001). Regarding specificity, no significant difference was observed between the two imaging methods (75.9% vs. 70.7%, *p* = 0.174). The negative predictive value, positive predictive value, and accuracy of PET/CT and MRI were 94.2% vs. 81.3%, 85.0% vs. 81.0%, and 88.0% vs. 81.1%, respectively (Table 1). The area under the curve (AUC) of PET/CT was higher than that of MRI (0.863 vs. 0.796, *p* < 0.05). Notably, 14.4% (66/460) of lymph nodes had discrepancies between the two imaging tests. Among them, PET/CT showed true positives in 22 lymph nodes that were mistakenly diagnosed as negative lymph nodes by MRI and true negatives in 27 lymph nodes misdiagnosed as positive lymph nodes by MRI. Nonetheless, 17 lymph nodes were wrongly diagnosed as positive lymph nodes by PET/CT according to histopathology.

**Table 1:** Performance of PET/CT versus MRI for diagnosing 460 biopsied cervical lymph nodes of 336 patients

Imaging methods	TP	TN	FP	FN	Sensitivity (%)	*p*	Specificity (%)	*p*	PPV (%)	*p*	NPV (%)	*p*	Accuracy (%)	*p*
					95% CI		95% CI		95% CI		95% CI		95% CI	
PET/CT	260	145	46	9	96.7 (93.5–98.4)		75.9 (69.1–81.7)		85.0 (80.4–88.7)		94.2 (88.9–97.1)		88.0 (84.7–90.9)	
MRI	238	135	56	31	88.5 (83.9–91.9)		70.7 (63.6–76.9)		81.0 (75.9–85.2)		81.3 (74.4–86.8)		81.1 (77.2–84.6)	
PET/CT vs. MRI						< 0.001		0.174		0.191		0.001		< 0.001

Abbreviations: *FP*, false positive; *FN*, false negative; *NPV*, negative predictive value; *MRI*, magnetic resonance imaging; *PET/CT*, [18F]-fluorodeoxyglucose positron emission tomography/computed tomography; *PPV*, positive predictive value; *TP*, true positive; *TN*, true negative

To evaluate whether the staging is altered by PET/CT or MRI and subsequently affects the prognosis, cohort B included 1093 patients who received pretreatment PET/CT and MRI. Their median age was 45 (range, 12–79) years, men accounted for 71.9%, and 819 (74.9%) patients had EBV DNA less than 4000 copies/mL (Table 2). With a median follow-up time of 50 (range, 1–118) months, 48 (4.4%) patients died, 142 (13.9%) patients suffered from treatment failure, and 62 (5.7%) patients had distant metastasis. The 5-year OS, FFS, DMFS, and LRRFS rates were 96.0%, 85.6%, 93.6%, and 92.9%, respectively.

**Table 2:** Baseline characteristics of patients with T3N1M0 in the primary cohort B, cohort C, and PSM cohort

	Primary cohort			PSM cohort		
	Cohort B: PET/CT + MRI (*N* = 1093)	Cohort C: MRI (*N* = 1377)	*p*	PET/CT + MRI (*N* = 954)	MRI (*N* = 954)	*p*
	*N* (%)	*N* (%)		*N* (%)	*N* (%)	
Age			0.021			0.911
Median(range)	45 (12–79)	47 (13–81)		46 (12–79)	46 (15–78)	
< 55	867 (79.3)	1037 (75.3)		747 (78.3)	750 (78.6)	
≥ 55	226 (20.7)	340 (24.7)		207 (21.7)	204 (21.4)	
Sex			0.362			0.754
Male	786 (71.9)	966 (70.2)		709 (74.3)	702 (73.6)	
Female	307 (28.1)	411 (29.8)		245 (25.7)	252 (26.4)	
Albumin (g/L)			0.014			0.445
< 40	51 (4.7)	98 (7.1)		48 (5.0)	40 (4.2)	
≥ 40	1042 (95.3)	1279 (92.9)		906 (95.0)	914 (95.8)	
Hemoglobin (g/L)			0.163			0.849
< 120	61 (5.6)	97 (7.0)		57 (6.0)	60 (6.3)	
≥ 120	1032 (94.4)	1280 (93.0)		897 (94.0)	894 (93.7)	
LDH (U/L)			0.949			0.904
< 250	1050 (96.1)	1321 (95.9)		919 (96.3)	917 (96.1)	
≥ 250	43 (3.9)	56 (4.1)		35 (3.7)	37 (3.9)	
EBV DNA (copy/mL)			0.721			1.000
< 4000	819 (74.9)	1022 (74.2)		712 (74.6)	711 (74.5)	
≥ 4000	274 (25.1)	355 (25.8)		242 (25.4)	243 (25.5)	
Lymph node			< 0.001			0.882
Retropharyngeal lymph node	299 (27.4)	656 (47.6)		295 (30.9)	299 (31.3)	
Cervical lymph node	794 (72.6)	721 (52.4)		659 (69.1)	655 (68.7)	
Treatment			0.162			0.578
CCRT	489 (44.7)	584 (42.4)		407 (42.7)	422 (44.2)	
IC + CCRT	349 (31.9)	418 (30.4)		311 (32.6)	297 (31.1)	
RT	116 (10.6)	163 (11.8)		106 (11.1)	93 (9.7)	
IC + RT	139 (12.7)	212 (15.4)		130 (13.6)	142 (14.9)	
Smoking			0.266			0.610
Yes	296 (27.1)	402 (29.2)		261 (27.4)	272 (28.5)	
No	797 (72.9)	975 (70.8)		693 (72.6)	682 (71.5)	
Drinking			0.387			0.749
Yes	175 (16.0)	202 (14.7)		141 (14.8)	147 (15.4)	
No	918 (84.0)	1175 (85.3)		813 (85.2)	807 (84.6)	
History			1.000			0.323
Yes	107 (9.8)	135 (9.8)		74 (7.8)	87 (9.1)	
No	986 (90.2)	1242 (90.2)		880 (92.2)	867 (90.9)	

Abbreviations: *CCRT*, concurrent chemoradiotherapy; *EBV*, Epstein-Barr virus; *IC*, induction chemotherapy; *LDH*, serum lactate dehydrogenase; *MRI*, magnetic resonance imaging; *PSM*, propensity scoring matching; *PET/CT*, [18F]-fluorodeoxyglucose positron emission tomography/computed tomography; *RT*, radiotherapy

Based on PET/CT alone, 664 of 1093 patients in cohort B were staged as T3N1M0. All of the patients were consistently staged with T3 by MRI, whereas 3.2% (20/664), 83.1% (526/664), 13.4% (85/664), and 5.2% (33/664) of patients were diagnosed with N0, N1, N2, and N3 by MRI alone. However, remarkably, no significant differences in OS, FFS, LRRFS, or DMFS were observed among these N0, N1, N2, and N3 patients staged by MRI (*p* = 0.68, *p* = 0.68, *p* = 0.61, and *p* = 0.96, respectively; Supplementary Fig. 2).

Based on the MRI criteria alone, 599 of 1093 patients in cohort B were diagnosed with T3N1M0. All of these patients were also staged as N1 by PET/CT. Nonetheless, 12.2% (73/599) and 87.8% (526/599) of patients were classified as T2 and T3 retrospectively by PET/CT alone. The survival rates were not significantly different between T2 and T3 patients staged by PET/CT (*p* = 0.72 for OS, *p* = 0.85 for FFS, *p* = 0.93 for LRRFS, and *p* = 0.65 for DMFS; Supplementary Fig. 3).

### Prolonged survival rates of patients staged by PET/CT vs. MRI

To determine whether the advantage of PET/CT in diagnosis can contribute to survival differences, cohort C, in which patients underwent MRI only, was compared with cohort B. As shown in Table 2, the baseline characteristics between PET/CT plus MRI and MRI alone were compared. However, the results showed that there was an imbalance in age, lymph node location, and albumin between the two groups (*p* = 0.021, *p* = 0.014, and *p* < 0.001, respectively). After PSM at a ratio of 1:1, no imbalanced variables were observed between the two groups. Of 1908 patients who were included in the PSM cohort, 485 (25.4%) patients had EBV DNA greater than 4000 copies/mL. With a median follow-up period of 52 (1–151) months, 132 (6.9%) patients died, 165 (6.5%) patients developed distant metastasis, 211 (11.1%) patients suffered from locoregional relapse, and 93 (4.9%) patients had nodal recurrences. The 5-year OS, FFS, DMFS, and LRRFS rates were 93.0%, 78.6%, 90.9%, and 87.2%, respectively.

**Fig. 2:**
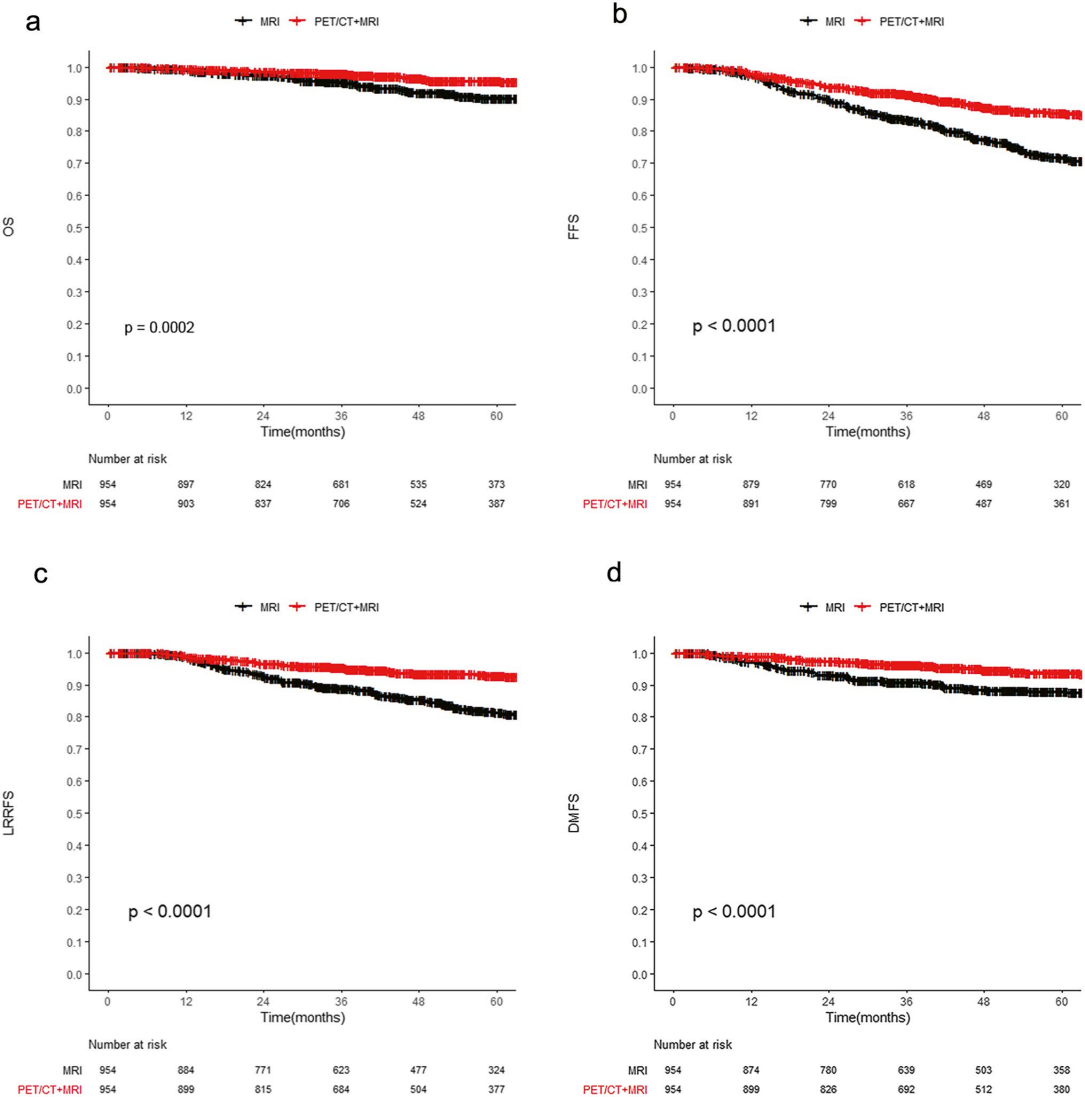
Survival curves comparing PET/CT + MRI with MRI alone in the PSM cohort: OS (**a**), FFS (**b**), LRRFS (**c**), and DMFS (**d**). Abbreviations: DMFS, distant metastasis-free survival; FFS, failure-free survival; LRRFS, locoregional relapse-free survival; MRI, magnetic resonance imaging; OS, overall survival; PET/CT, [18F]-fluorodeoxyglucose positron emission tomography with computed tomography; PSM, propensity scoring matching

In the survival analysis, patients who underwent both PET/CT and MRI had better OS than those who underwent MRI alone (5-year OS, 95.7% vs. 90.4%, *p* < 0.001). In terms of FFS, DMFS, and LRRFS, patients receiving both PET/CT and MRI also had better outcomes than those receiving MRI alone (5-year FFS, 85.7% vs. 71.7%, *p* < 0.001; 5-year DMFS, 93.9% vs. 87.9%, *p* < 0.001; and 5-year LRRFS, 93% vs. 81.4%, *p* < 0.001; Fig. 2). The univariate analysis is presented in Supplementary Table 2. As shown in Supplementary Table 3, multivariate analysis indicated that the application of PET/CT was an independent favorable prognostic factor for OS (hazard ratio [HR] = 0.5, 95% confidence interval [CI] 0.3–0.7, *p* < 0.001), FFS (HR = 0.5, 95% CI 0.4–0.6, *p* < 0.001), DMFS (HR = 0.4, 95% CI 0.3–0.6, *p* < 0.001), and LRRFS (HR = 0.4, 95% CI 0.3–0.5, *p* < 0.001).

Subgroup analysis was conducted for patients with EBV DNA less than 4000 copies/mL in the PSM cohort. In this subgroup, 1423 patients were eligible, among which 712 patients received both PET/CT and MRI. As shown in Supplementary Fig. 4, patients undergoing both PET/CT and MRI had a survival benefit for OS (5-year OS, 96.5% vs. 91.4%, *p* = 0.0012), FFS (5-year FFS, 86.1% vs. 75.4%, *p* < 0.001), DMFS (5-year DMFS, 93.7% vs. 90.9%, *p* < 0.001), and LRRFS (5-year LRRFS, 92.9% vs. 84.2%, *p* = 0.004) compared with those undergoing MRI alone. The univariate analysis is detailed in Supplementary Table 4. In the multivariable analysis (see Supplementary Table 5), the addition of PET/CT was also an independent factor for OS (HR = 0.5, 95% CI 0.3–0.8, *p* = 0.001), FFS (HR = 0.5, 95% CI 0.4–0.7, *p* < 0.001), DMFS (HR = 0.6, 95% CI 0.4–0.8, *p* = 0.004), and LRRFS (HR = 0.5, 95% CI 0.3–0.6, *p* < 0.001).

### Guiding individualized induction chemotherapy

In cohort B, 838 patients who received concurrent chemoradiotherapy with or without induction chemotherapy were selected for cohort D. However, there were significant differences in baseline characteristics between the two treatment modalities (see Table 3). After PSM at a 1:1 ratio, 698 patients were included in this now well-balanced cohort. In PSM cohort D, the median age was 46 years old (range, 13–73), 132 (18.9%) patients had lymph node necrosis, and patients with grade 0, 1, 2, and 3 radiologic extranodal extension accounted for 48.1% (336/698), 18.8% (131/698), 20.9% (146/698), and 12.2% (85/698), respectively. With a median follow-up period of 50 (1–118) months, 117, 50, 45, and 35 patients had treatment failure, locoregional relapse, distant metastasis, and death, respectively. The 5-year FFS, LRRFS, DMFS, and OS rates were 82.0%, 92.6%, 92.8%, and 95.0%, respectively. Interestingly, univariate analysis (Supplementary Table 6) and multivariable analysis indicated that an SUVmax-N higher than 9.35, together with nodal necrosis and extranodal extension infiltrating adjacent structures, had prognostic significance for FFS (*p* < 0.001, *p* = 0.002, and *p* = 0.002, respectively, Supplementary Table 7). The radiologic score was thus developed based on the number of the three factors. Patients with higher radiologic scores had lower FFS (*p* < 0.001, Supplementary Fig. 5). Thus, patients with one or more risk factors were classified into the high-risk group (radiologic score > 0, *n* = 454), while patients with no risk factors were stratified into the low-risk group (radiologic score = 0, *n* = 244). The survival curves showed that patients in the high-risk group had lower FFS, DMFS, LRRFS, and OS than those in the lower-risk group (all *p* < 0.05, Supplementary Fig. 6). The radiologic score model had a higher C-index than the model with sex and EBV DNA (0.72 [95% CI: 0.65–0.78] vs. 0.56 [95% CI: 0.49–0.63], *p* < 0.001).

**Table 3:** Baseline characteristics of patients in the primary cohort D and PSM cohort D

	Primary cohort D			PSM cohort D		
	CCRT (*N* = 489)	IC + CCRT (*N* = 349)	*p*	CCRT (*n* = 349)	IC + CCRT (*n* = 349)	*p*
	*n* (%)	*n* (%)		*n* (%)	*n* (%)	
Sex			0.617			0.511
Female	152 (31.1)	102 (29.2)		111 (31.8)	102 (29.2)	
Male	337 (68.9)	247 (70.8)		238 (68.2)	247 (70.8)	
Age			0.297			0.306
< 55	394 (80.6)	270 (77.4)		282 (80.8)	270 (77.4)	
≥ 55	95 (19.4)	79 (22.6)		67 (19.2)	79 (22.6)	
Albumin (g/L)			0.336			0.704
< 40	15 (3.1)	16 (4.9)		13 (3.7)	16 (4.9)	
≥ 40	474 (96.9)	333 (95.4)		336 (96.3)	333 (95.4)	
Hemoglobin (g/L)			0.786			0.658
< 120	14 (2.9)	12 (3.4)		9 (2.6)	12 (3.4)	
≥ 120	475 (97.1)	337 (96.6)		340 (97.4)	337 (96.6)	
LDH(U/L)			0.878			0.066
< 250	462 (94.5)	328 (94.0)		339 (97.1)	328 (94)	
≥ 250	27 (5.5)	21 (6.0)		10 (2.9)	21 (6.0)	
EBV DNA (copy/mL)			0.001			0.073
< 2000	341 (69.7)	203 (58.2)		227 (65.0)	203 (58.2)	
≥ 2000	148 (30.3)	146 (41.8)		122 (35.0)	146 (41.8)	
Lymph node			0.015			0.540
CLN	379 (77.5)	295 (84.5)		288 (82.5)	295 (84.5)	
RLN	110 (22.5)	54 (15.5)		61 (17.5)	54 (15.5)	
Smoking			0.431			0.729
Yes	135 (27.6)	87 (24.9)		92 (26.4)	87 (24.9)	
No	354 (72.4)	262 (75.1)		257 (73.6)	262 (75.1)	
Drinking			1.000			1.000
Yes	81 (16.6)	58 (16.6)		59 (16.9)	58 (16.6)	
No	408 (83.4)	291 (83.4)		290 (83.1)	291 (83.4)	
History			0.117			0.076
Yes	55 (11.2)	27 (7.7)		42 (12.0)	27 (7.7)	
No	434 (88.8)	322 (92.3)		307 (88.0)	322 (92.3)	
Nodal necrosis			0.034			0.384
Yes	71 (14.5)	71 (20.3)		61 (17.5)	71 (20.3)	
No	418 (85.5)	278 (79.7)		217 (82.5)	278 (79.7)	
Minimal axial diameter(cm)			0.001			0.245
< 0.95	186 (38.0)	95 (27.2)		110 (31.5)	95 (27.2)	
≥ 0.95	303 (62.0)	254 (72.8)		239 (68.5)	254 (72.8)	
Maximal axial diameter(cm)			< 0.001			0.148
< 1.35	208 (42.5)	106 (30.4)		125 (35.8)	106 (30.4)	
≥ 1.35	281 (57.5)	243 (69.6)		224 (64.2)	243 (69.6)	
SUVmax-T			0.318			0.929
< 9.25	131 (26.8)	82 (23.5)		80 (22.9)	82 (23.5)	
≥ 9.25	358 (73.2)	267 (76.5)		269 (77.1)	267 (76.5)	
SUVmax-N			< 0.001			0.590
< 9.35	286 (58.5)	138 (39.5)		146(41.8)	138 (39.5)	
≥ 9.35	203 (41.5)	211 (60.5)		203 (58.2)	211 (60.5)	
rENE			0.020			0.240
Grade 0	261 (53.4)	164 (47.0)		172 (49.3)	164 (47.0)	
Grade 1	96 (19.6)	61 (17.5)		70 (20.1)	61 (17.5)	
Grade 2	91 (18.6)	73 (20.9)		73 (20.9)	73 (20.9)	
Grade 3	41 (8.4)	51 (14.6)		34 (9.7)	51 (14.6)	

Abbreviations: *CCRT*, concurrent radiochemotherapy; *CLN*, cervical lymph node; *EBV*, Epstein-Barr virus; *IC*, induction chemotherapy; *LDH*, serum lactate dehydrogenase; *PSM*, propensity scoring matching; *rENE*, radiologic extranodal extension; *RLN*, retropharyngeal lymph node; *SUVmax-N*, the maximal standardized uptake value of lymph node; *SUVmax-T*, the maximal standardized uptake value of primary tumor

**Fig. 3:**
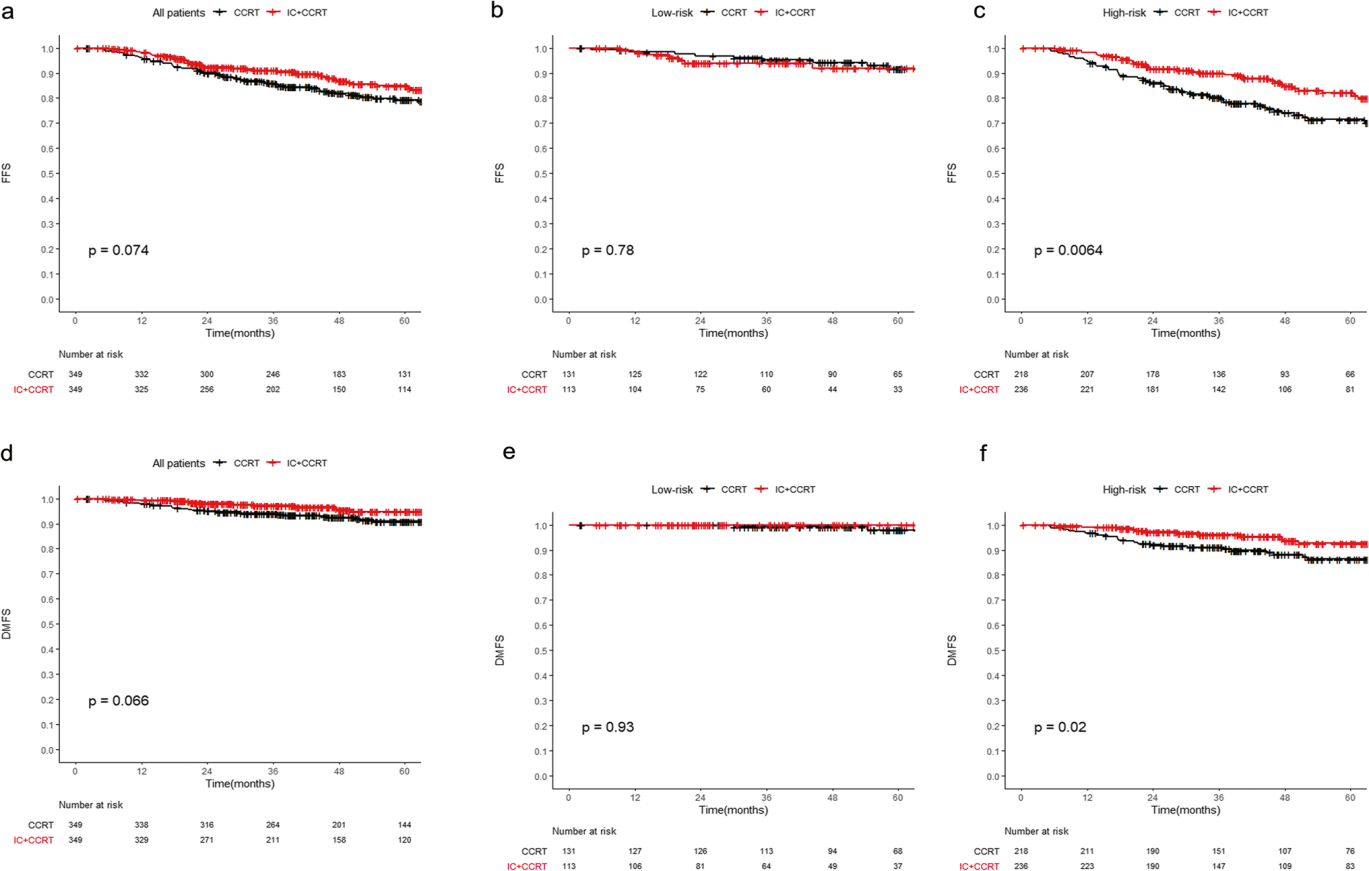
Kaplan–Meier FFS curves comparing IC + CCRT and CCRT alone in the whole PSM cohort D (**a**), low-risk group (**b**), and high-risk group (**c**) and DMFS curves in the whole PSM cohort D (**d**), low-risk group (**e**), and high-risk group (**f**). Abbreviations: CCRT, concurrent chemoradiotherapy; DMFS, distant metastasis-free survival; FFS, failure-free survival; IC, induction chemotherapy; PSM, propensity scoring matching

For all of the patients, induction chemotherapy showed no survival benefit (*p* = 0.78, Fig. 3). However, in the high-risk group stratified by radiologic score, patients receiving induction chemotherapy plus concurrent chemoradiotherapy had a higher 5-year FFS than those receiving concurrent chemoradiotherapy alone (82.2% vs. 71.5%; *p* = 0.006, Fig. 3). After adjusting for covariates, multivariate analysis also confirmed that the addition of induction chemotherapy was an independent risk factor for FFS (HR: 0.5, 95% CI: 0.4–0.8, *p* = 0.003; Supplementary Table 8 and Supplementary Table 9). In contrast, no survival difference was observed between the two treatment modes in the low-risk group (*p* = 0.074, Fig. 3). The same conclusion was also reached for DMFS and RRFS. The detailed results for DMFS, LRRFS, and OS are shown in the supplementary Tables and Figures.

## Discussion

In this large cohort study, PET/CT was proven to be more accurate than MRI for diagnosing cervical lymph nodes as confirmed by histopathology. Accordingly, as accurate N staging more precisely revealed the true prognosis of patients, PET/CT plus MRI could identify T3N1M0 patients who had better survival outcomes than MRI-staged T3N1M0 patients, even if their EBV DNA was less than 4000 copies/mL. PET/CT-based SUVmax of lymph nodes together with nodal necrosis and extranodal extension involving adjacent structures could be used to build a radiologic score model and identify high-risk T3N1M0 patients who can benefit from the addition of induction chemotherapy.

In fact, this is not the first report of the advantage of PET/CT over MRI for diagnosing lymph nodes in patients with nasopharyngeal carcinoma. However, different from previous studies [4, 15], the 460 lymph nodes included in our study were pathologically confirmed instead of by clinical follow-up. Certainly, the mistakes and biased of mapping biopsied lymph nodes on PET/CT and MRI images cannot be absolutely avoided, although we included solitary lymph nodes, the largest lymph node, and other nodes that could be definitely located without any uncertainty according to the ultrasonic reports and graphs with detailed characteristics of the biopsied node, such as the exact size, level, extranodal extension, and distance from the skin, muscles, vein, artery, or other landmark structures. In addition, the findings of PET/CT being superior to MRI were also consistent with the results of studies in head and neck cancer [16]. Although the sensitivity of PET/CT we found in nasopharyngeal carcinoma (96.7%) was slightly higher than that of head and neck cancer (90.0%), the specificity of PET/CT was only 75.9% herein, lower than that of head and neck cancer (94.0%) [17] but still better than MRI (70.8%). Perhaps a new deep learning algorithm might be a good assistant to further improve its diagnostic performance. Notably, 18.6% (118/633) of PET/CT-diagnosed T3N1M0 cases were upstaged to T3N2-3M0 by MRI, while only 3.2% (20/633) of patients were downstaged to T3N0M0 by MRI, without any discrepancy in T3 staging. This also indicated the strong potential of overdiagnosis by MRI. The similar survival rates (Supplementary Fig. 2) across the misdiagnosed T3N0-3M0 patients by MRI but staged T3N1M0 by PET/CT again supported the higher possibility of getting closer to the true prognosis if staged by PET/CT in terms of treatment outcomes. Certainly, MRI-staged T3N1M0 patients were divided into T2N1M0 and T3N1M0 by PET/CT, but no survival differences were observed between T2 and T3; as no gold standard to confirm the T stage of either type of examination equipment was used, we failed to draw a firm conclusion for PET/CT versus MRI for the T stage of nasopharyngeal carcinoma. Reviewing previous studies [15, 18], MRI seemed to be more accurate than PET/CT for diagnosing the involvement of local structures. Therefore, a combination of PET/CT and MRI may be the best recommendation for diagnosing and staging treatment-naïve nasopharyngeal carcinoma.

To test whether the diagnostic advantages of PET/CT can benefit treatment outcomes, we directly compared two cohorts of T3N1M0 patients staged by PET/CT plus MRI or MRI alone. The significantly higher survival rates of patients with PET/CT plus MRI, regardless of the EBV DNA load, supported our supposition. Obviously, PET/CT as an examination test cannot directly alter the final treatment outcomes by itself, but several prospective studies reported that adding PET/CT to conventional work-ups could provide additional information and may change management approaches in 15.7–33.8% of head and neck cancer patients [16, 19, 20]. Given the retrospective design of our study, the magnitude of actual changes in treatment choices before and after the application of PET/CT in these patients was not available. Although PET/CT is recommended at primary staging for patients with initially unknown primary cancer, enlarged lymph nodes at multiple or lower levels, locoregionally advanced cancer, or ambiguous lymph nodes or metastases by conventional imaging [3, 21], patient requests and doctor preferences should also be considered in clinical practice. Certainly, the potential bias related to financial constraints is hard to eliminate in retrospective studies, even in randomized clinical trials. Patients with good financial support tend to receive more advanced chemotherapy regimens, better supportive treatment, and more regular surveillance. On the other hand, we aimed to investigate whether PET/CT could predict the survival rate of patients and accordingly identify high-risk patients to receive more intensive treatment. In a cohort of PET/CT- and MRI-staged T3N1M0 patients, induction chemotherapy showed no survival benefit for all patients, as observed in prior studies [9]. Previously reported EBV DNA load- and sex-guided risk stratification [10] did not work here, possibly because of its poor specificity and generalization. In contrast, SUVmax-N, nodal necrosis, and extranodal extension involved with adjacent structures remained highly prognostic. In fact, these were previously reported [12, 13, 22], which indicated the potential for good generalization.

The radiologic score model based on the three characteristics showed a significantly (*p* < 0.001) higher C-index (0.72) than the model based on EBV DNA and sex [10] (C-index = 0.56) in the risk stratification. In addition, the radiologic score model selected high-risk patients who could benefit from the addition of induction chemotherapy. As shown in our study, the 5-year FFS rate of the high-risk T3N1M0 patients was similar to those of more advanced patients included in clinical trials of induction chemotherapy [23]. Thus, it is not unreasonable that the high-risk T3N1M0 patients had significantly improved survival outcomes when receiving induction chemotherapy followed by concurrent chemoradiotherapy.

Based on 460 biopsied cervical lymph nodes, we firmly concluded there is an advantage of PET/CT for diagnosing lymph nodes. In a cohort of T3N1M0 patients, the interference of covariate factors including nodal size, nodal level, nodal laterality, and T stage was completely excluded; we proved the survival benefit was due to the accurate diagnosis by PET/CT and found the way to guide individualized induction chemotherapy for T3N1M0 patients by applying a radiologic score model based on PET/CT and MRI.

## Supplementary Information

Below is the link to the electronic supplementary material.

Supplementary file1 (PDF 1921 KB)

## Data Availability

The data and materials are available from the corresponding author on reasonable request.
