# Supplementary material for: Benefit of [18F]-FDG PET/CT for treatment-naïve nasopharyngeal carcinoma
Source: Eur J Nucl Med Mol Imaging. 2021 Sep 1;49(3):980–91. doi: 10.1007/s00259-021-05540-8 (PMC8803713; doi:10.1007/s00259-021-05540-8)
Supplement: Supplementary file 1 — Supplementary file1 (PDF 1921 KB) [file 259_2021_5540_MOESM1_ESM.pdf]

Title: Benefit of [18F]-FDG PET/CT for treatment-naïve nasopharyngeal carcinoma

Journal name: European Journal of Nuclear Medicine and Molecular Imaging

Author name: Shan-Shan Yang, MD [1†], Yi-Shan Wu, MD[2†], Wei-Chao Chen, MD[3†], Jun Zhang, MD[1], Su-Ming Xiao, MD [1], Bao-Yu Zhang, MD [1], Zhi-Qiao Liu, MD [1], En-Ni Chen, MD [1], Xu Zhang, MD [4*], Pu-Yun OuYang, MD [1*]and Fang-Yun Xie, MD [1*]

Correspondence to Prof. Fang-Yun Xie. Department of Radiation Oncology, Sun Yat-sen University Cancer Center, State Key Laboratory of Oncology in South China, Collaborative Innovation Center for Cancer Medicine, Guangdong Key Laboratory of Nasopharyngeal Carcinoma Diagnosis and Therapy, Guangzhou, China;

E-mail: xiefy@sysucc.org.cn

MRI protocol

All patients underwent head and neck MR imaging with a 1.5- or 3.0-T system (Signa CV/i, GE HealthCare, Chalfont St Giles, United Kingdom). The area from the suprasellar cistern to the inferior margin of the sternal end of the clavicle was scanned. T1-weighted fast spin-echo images in the axial, coronal, and sagittal planes (repetition time: 500–600 ms, echo time: 10–20 ms, and field of view: 22 cm) and T2-weighted fast spin-echo MR images in the axial plane (repetition time: 4000–6000 ms, echo time: 95–110 ms, and field of view: 22 cm) were obtained before injection of contrast material. Spin-echo T1-weighted axial and sagittal sequences and spin-echo T1-weighted fat-suppressed coronal sequences were performed after intravenous Gd-DTPA (Magnevist; Bayer Schering Pharma AG, Germany) injection at a dose of 0.1 mmol/kg.

PET/CT protocol

[18F]-FDG PET/CT scans were conducted using a Discovery ST-16 (GE Medical Systems, Milwaukee, WI, USA). The scan range was from the vertex to the upper thigh according to a standard whole-body acquisition protocol. Patients needed to fast for 6 hours before injecting [18F]-FDG. Then, imaging was performed approximately 45–60 mins after injection of 3.7 Mbq/kg of body weight (0.1 mCi/kg) of [18F]-FDG. Next, a low-dose multislice CT scan was obtained using a 16-slice multidetector scanner (parameters: 180–250 mA, 140 kV, pitch 1.375 mm, and slice thickness 3.75 mm) with shallow breathing. A standard whole-body PET scan was required in 2D mode with an acquisition time of 3 mins per bed position (six-eight bed positions) covering the same field as the CT scan. The acquired data were reconstructed using the ordered subset expectation maximization iterative algorithm (OSEM). Finally, the data were transferred to a workstation (AW Server 2.0; GE Health care) for processing and interpretation. The standard uptake value (SUV) was calculated using the body weight.

Supplementary Fig. 1 The flowchart of included patients.

Abbreviations: CCRT, concurrent chemoradiotherapy; IC, induction chemotherapy; NPC, nasopharyngeal carcinoma; MRI, magnetic resonance imaging; PET/CT, [18F]-fluorodeoxyglucose positron emission tomography with computed tomography.

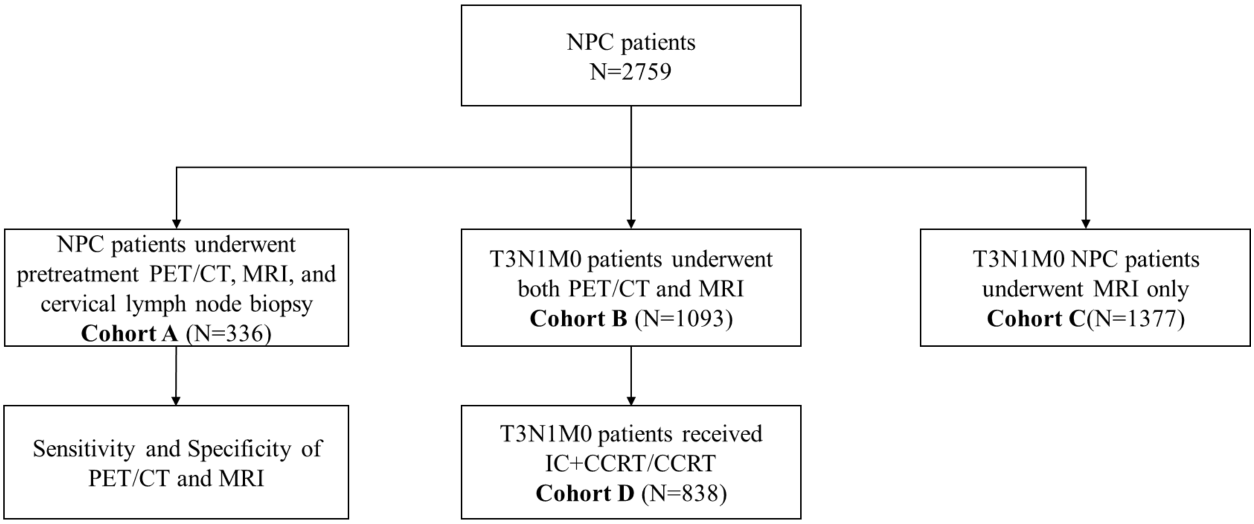

Supplementary Fig. 2 Kaplan–Meier survival curves stratified by MRI-based N stage in T3N1M0 patients staged by PET/CT: (a) OS, (b) FFS, (c) LRRFS, and (d) DMFS.

Abbreviations: DMFS, distant metastasis-free survival; FFS, failure-free survival; LRRFS, locoregional relapse-free survival; MRI, magnetic resonance imaging; OS, overall survival; PET/CT, [18F]-fluorodeoxyglucose positron emission tomography with computed tomography.

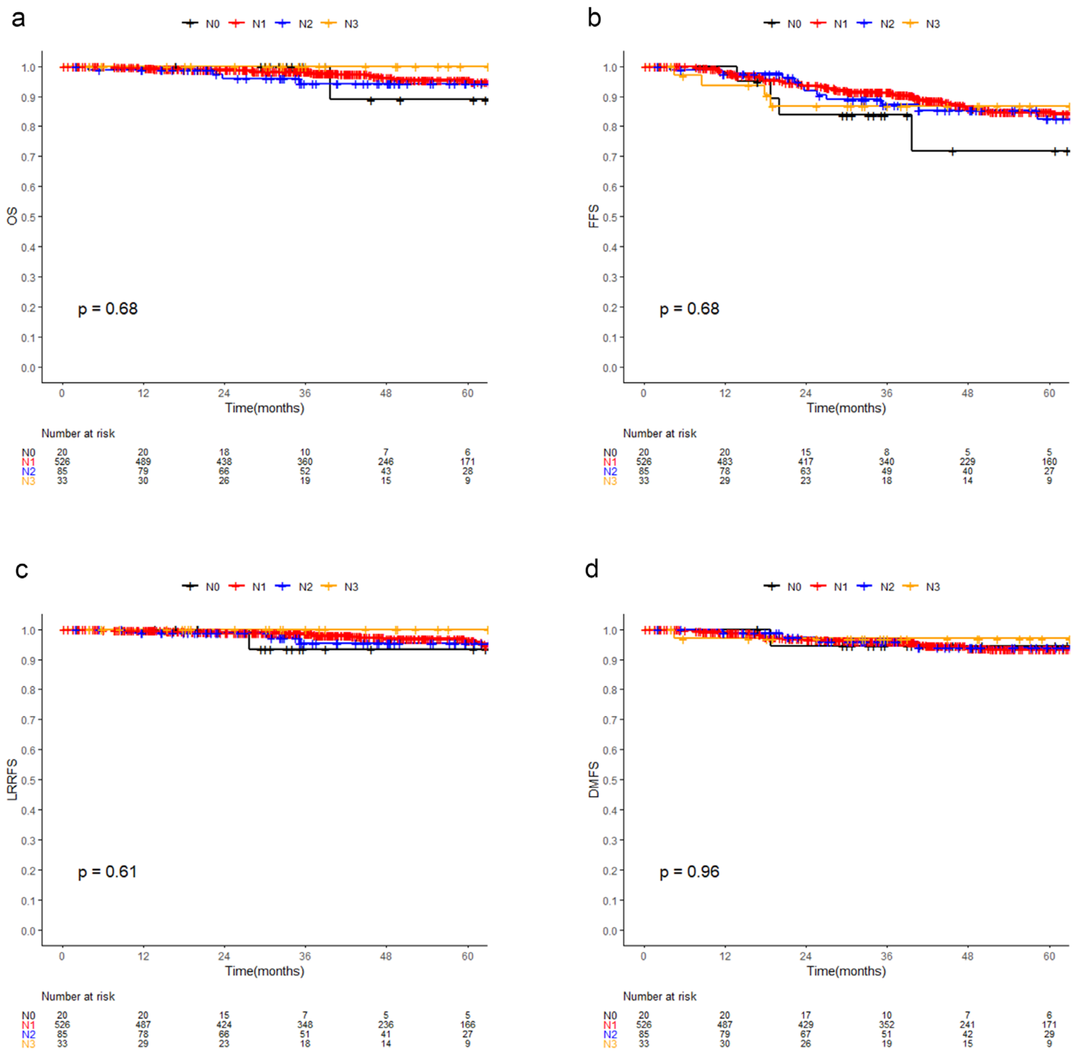

Supplementary Fig. 3 Kaplan–Meier survival curves stratified by PET/CT-based T stage in T3N1M0 patients staged by MRI: (a) OS, (b) FFS, (c) LRRFS, and (d) DMFS.

Abbreviations: DMFS, distant metastasis-free survival; FFS, failure-free survival; LRRFS, locoregional relapse-free survival; MRI, magnetic resonance imaging; OS, overall survival; PET/CT, [18F]-fluorodeoxyglucose positron emission tomography with computed tomography.

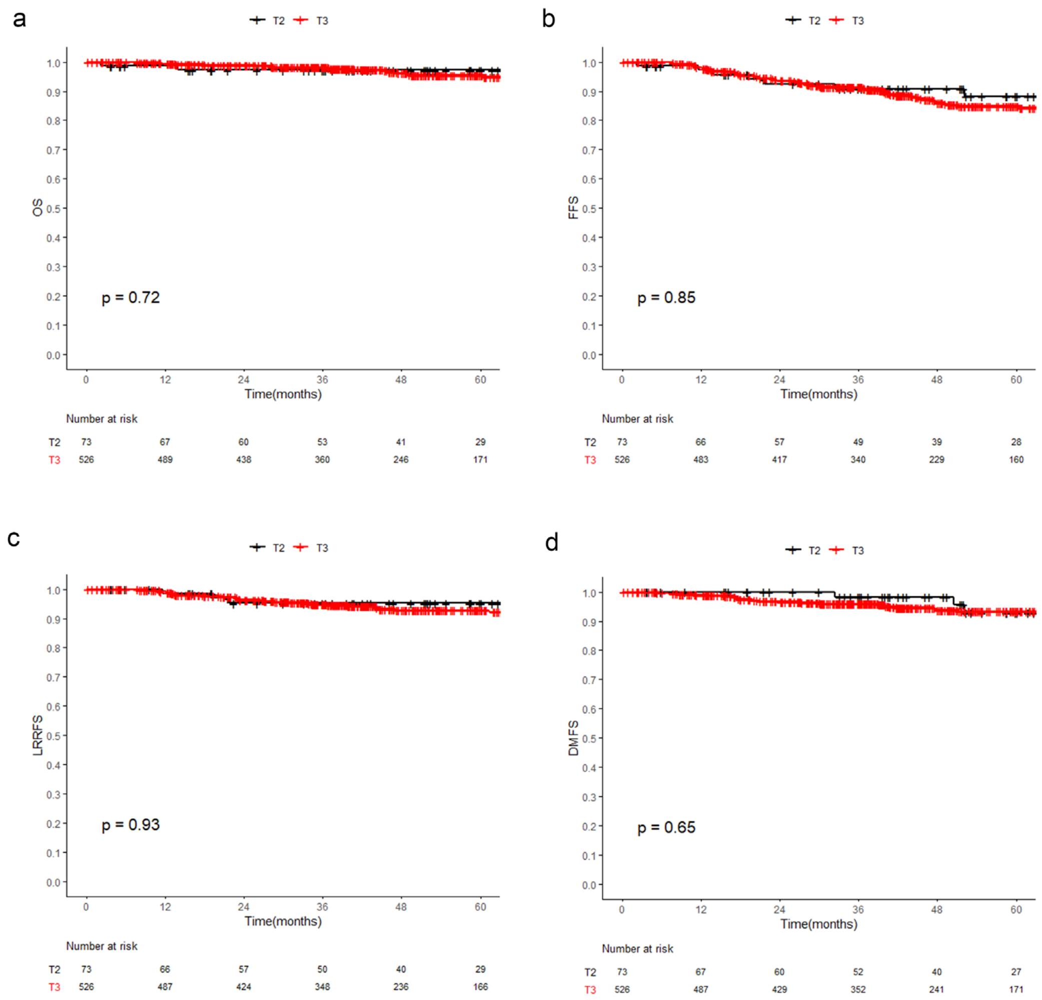

Supplementary Fig. 4 Survival curves of patients with EBV-DNA lower than 4000 copies/ml in the PSM cohort: (a) OS, (b) FFS, (c) LRRFS, and (d) DMFS.

Abbreviations: DMFS, distant metastasis-free survival; EBV, Epstein Barr virus; FFS, failure-free survival; LRRFS, locoregional relapse-free survival; MRI, magnetic resonance imaging; OS, overall survival; PET/CT, [18F]-fluorodeoxyglucose positron emission tomography with computed tomography; PSM, propensity scoring matching.

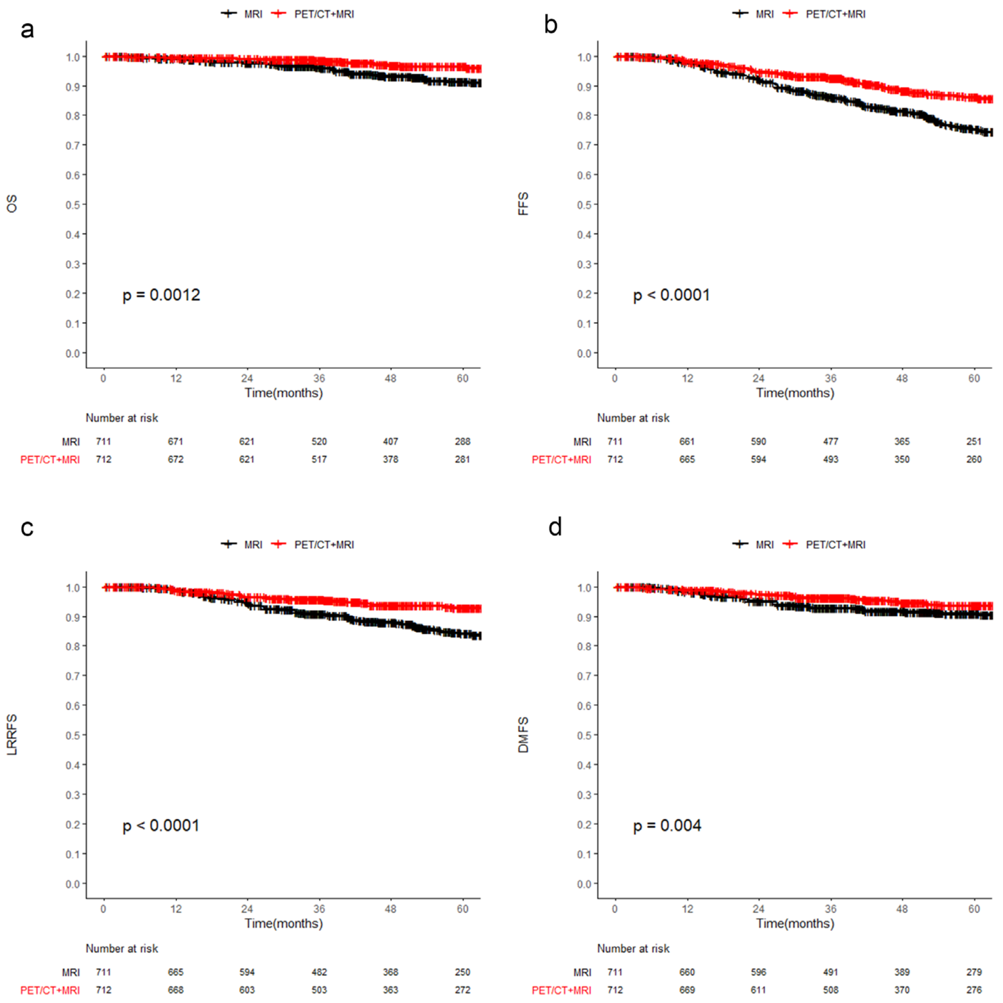

Supplementary Fig. 5 Survival curves of risk scores for FFS (a) and DMFS (b). Abbreviations: DMFS, distant metastasis-free survival; FFS, failure-free survival.

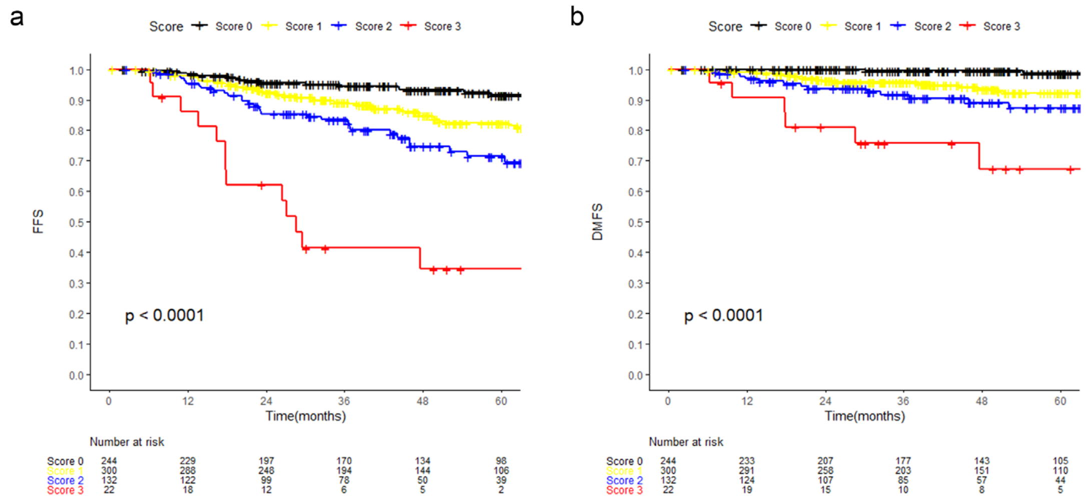

Supplementary Fig. 6 Survival curves of different risk groups for FFS (a), DMFS (b), LRRFS (c) and OS (d).

Abbreviations: DMFS, distant metastasis-free survival; FFS, failure-free survival; OS, overall survival; LRRFS, regional relapse-free survival.

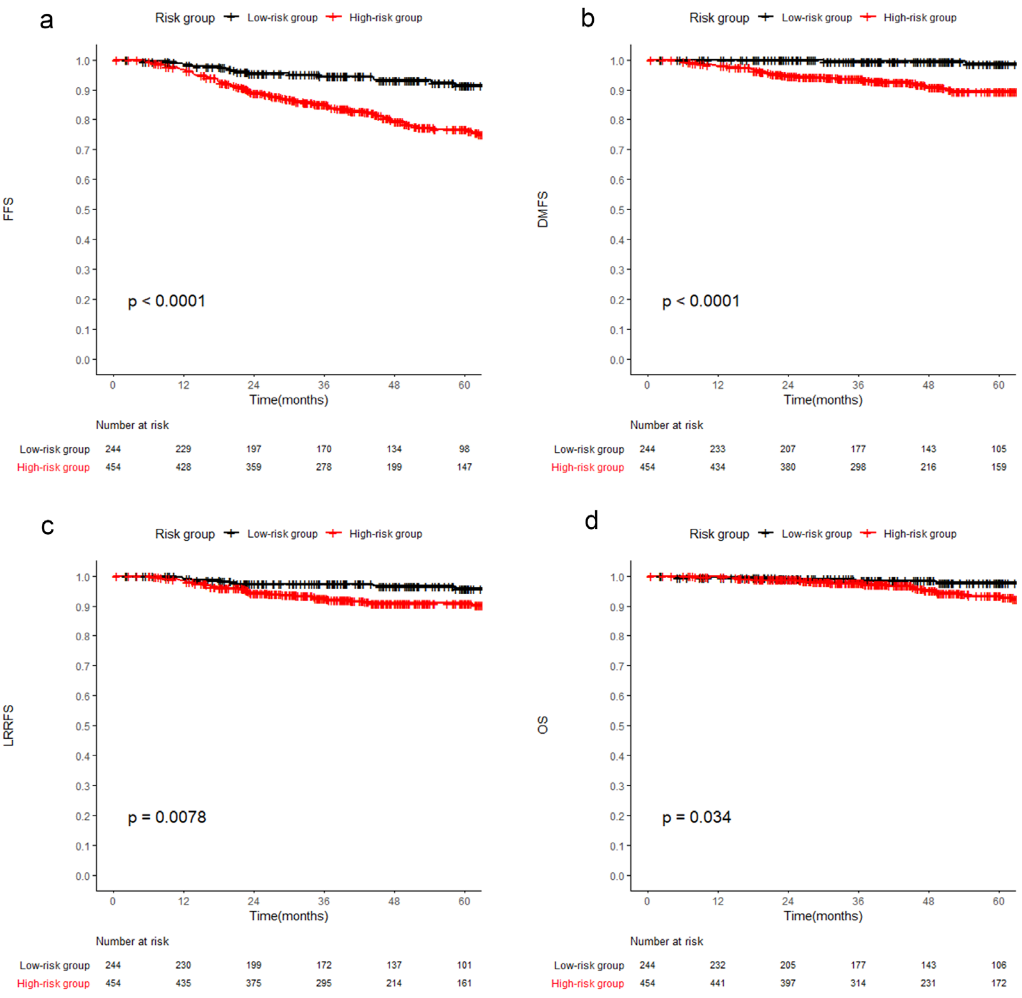

Supplementary Table 1. Baseline characteristics of patients receiving biopsy (Cohort A).

Characteristic		Cohort A (N=336)
		N (%)
Age		
	Median (Range)	45.5(15-74)
	<55	268(79.8)
	≥55	68(20.2)
Sex		
	Male	248(73.8)
	Female	88(26.2)
EBV-DNA		
	<4000	251(74.7)
	≥4000	85(25.3)
Treatment		
	CCRT	145(43.2)
	IC+CCRT	119(35.4)
	RT	21(6.3)
	IC+RT	24(7.1)
	Palliative care	27(8.0)
PET/CT		
	Yes	336(100)
	No	-
T stage		
	T1	17(5.0)
	T2	65(19.4)
	T3	211(62.8)
	T4	43(12.8)
N stage		
	N0	7(2.1)
	N1	259(77.1)
	N2	43(12.8)
	N3	27(8.0)
M stage		
	M0	309(92.0)
	M1	27(8.0)
TNM stage		
	I	3(0.9)
	II	57(17.0)
	III	195(58.0)
	IV	81(24.1)

Abbreviations: CCRT, concurrent chemoradiotherapy; EBV, Epstein Barr virus; IC, induction chemotherapy; PET/CT, [18F]-fluorodeoxyglucose positron emission tomography/computed tomography; RT, radiotherapy.

Supplementary Table 2. Univariate analysis of patients in the PSM cohort (N=1908).

	OS		FFS		DMFS		LRRFS	
	HR (95% CI)	p	HR (95% CI)	p	HR (95% CI)	p	HR (95% CI)	p
Age (≥55 vs. <55)	2.9 (2.0-4.1)	<0.001	1.4 (1.1-1.7)	0.012	1.3 (0.9-1.8)	0.150	0.8 (0.5-1.1)	0.180
Sex (Male vs. Female)	1.0 (0.7-1.5)	0.870	1.4 (1.1-1.8)	0.008	1.6 (1.1-2.4)	0.018	1.2 (0.9-1.7)	0.200
Hemoglobin (≥120 vs. <120)	1.3 (0.6-3.1)	0.480	1.4 (0.9-2.3)	0.170	2.6 (0.9-7.1)	0.056	1.2 (0.7-2.3)	0.500
Albumin (≥40 vs. <40)	0.4 (0.2-0.6)	<0.001	0.7 (0.4-1.0)	0.069	0.9 (0.4-1.8)	0.680	1.3 (0.6-2.8)	0.490
LDH (≥250 vs. <250)	1.6 (0.8-3.5)	0.200	1.8 (1.2-2.7)	<0.001	1.6 (0.8-3.1)	0.170	1.9 (1.1-3.4)	0.022
EBV DNA (≥4000 vs. <4000)	1.5 (1.0-2.2)	0.027	1.5 (1.2-1.9)	<0.001	1.8 (1.3-2.5)	<0.001	1.5 (1.1-2.0)	0.004
Lymph Node (CLN vs. RLN)	1.6 (1.1-2.4)	0.028	1.3 (1.1-1.7)	0.016	1.9 (1.3-2.8)	<0.001	1.2 (0.9-1.6)	0.250
PET/CT (Yes vs. No)	0.5 (0.4-0.7)	<0.001	0.5 (0.4-0.6)	<0.001	0.5 (0.3-0.6)	<0.001	0.4 (0.3-0.5)	<0.001
Treatment								
CCRT	Reference		Reference		Reference		Reference	
IC+CCRT	1.0 (0.7-1.5)	0.993	0.9 (0.7-1.1)	0.207	0.8 (0.6-1.2)	0.245	0.8 (0.6-1.1)	0.094
RT	1.7 (1.0-2.7)	0.038	1.1 (0.8-1.5)	0.671	1.0 (0.6-1.6)	0.939	0.6 (0.4-1.0)	0.063
IC+RT	0.6 (0.3-1.1)	0.122	0.8 (0.6-1.1)	0.149	0.8 (0.5-1.3)	0.330	0.9 (0.6-1.4)	0.675
Smoking (Yes vs. No)	1.2 (0.8-1.7)	0.390	1.1 (0.9-1.3)	0.620	0.9 (0.7-1.3)	0.750	1.0 (0.7-1.3)	0.860
Drinking (Yes vs. No)	1.3 (0.9-2.1)	0.210	1.2 (0.9-1.6)	0.160	1.2 (0.8-1.8)	0.440	1.0 (0.7-1.5)	0.840
History (Yes vs. No)	0.8 (0.4-1.6)	0.520	1.0 (0.7-1.5)	0.930	1.1 (0.7-1.9)	0.700	1.4 (0.9-2.2)	0.120

Abbreviations: CCRT, concurrent chemoradiotherapy; CI, confidence interval; CLN, cervical lymph node; DMFS, distant metastasis-free survival; EBV, Epstein Barr virus; FFS, failure-free survival; HR, hazard ratio; IC, induction chemotherapy; LDH, serum lactate dehydrogenase; LRRFS, locoregional relapse-free survival; OS, overall survival; PET/CT, [18F]-fluorodeoxyglucose positron emission tomography/computed tomography; PSM: propensity scoring matching; RLN, retropharyngeal lymph node; RT, radiotherapy.

Supplementary Table 3. Multivariable analysis in the PSM cohort (N=1908).

	OS		FFS		DMFS		LRRFS	
	HR (95% CI)	p	HR (95% CI)	p	HR (95% CI)	p	HR (95% CI)	p
Age (≥55 vs. <55)	2.7 (1.9-3.8)	<0.001						
Sex (Male vs. Female)			1.4 (1.1-1.8)	0.008	1.6 (1.1-2.4)	0.017		
Albumin (≥120 vs. <120)	0.4 (0.2-0.7)	0.002						
EBV DNA (≥4000 vs. <4000)			1.5 (1.2-1.8)	<0.001	1.6 (1.2-2.3)	0.003	1.5 (1.1-2.1)	0.004
Lymph Node (CLN vs. RLN)					1.7 (1.2-2.5)	0.006		
PET/CT (Yes vs. No)	0.5 (0.3-0.7)	<0.001	0.5 (0.4-0.6)	<0.001	0.4 (0.3-0.6)	<0.001	0.4 (0.3-0.5)	<0.001

Abbreviations: CI, confidence interval; CLN, cervical lymph node; DMFS, distant metastasis-free survival; EBV, Epstein Barr virus; FFS, failure-free survival; HR, hazard ratio; LRRFS, locoregional relapse-free survival; OS, overall survival; PET/CT, [18F]-fluorodeoxyglucose positron emission tomography/computed tomography; PSM: propensity scoring matching; RLN, retropharyngeal lymph node.

Supplementary Table 4. Univariate analysis of patients with EBV-DNA less than 4000 copies/ml in the PSM cohort (N=1423).

	OS		FFS		DMFS		LRRFS	
	HR (95% CI)	p	HR (95% CI)	p	HR (95% CI)	p	HR (95% CI)	p
Age (≥55 vs. <55)	3.3 (2.2-5.0)	<0.001	1.4 (1.0-1.8)	0.036	1.2 (0.8-2.0)	0.340	0.8 (0.5-1.3)	0.360
Sex (Male vs. Female)	0.9 (0.6-1.5)	0.790	1.2 (0.9-1.6)	0.160	1.4 (0.9-2.3)	0.140	1.1 (0.7-1.6)	0.730
Hemoglobin (≥120 vs. <120)	0.9 (0.4-2.3)	0.890	1.0 (0.6-1.8)	0.900	1.4 (0.5-3.9)	0.490	1.0 (0.5-2.0)	0.960
Albumin (≥40 vs. <40)	0.4 (0.2-0.9)	0.018	0.6 (0.4-1.0)	0.070	0.7 (0.3-1.6)	0.370	1.0 (0.4-2.2)	0.940
LDH (≥250 vs. <250)	1.1 (0.3-4.6)	0.870	1.5 (0.8-3.1)	0.250	1.4 (0.4-4.4)	0.570	1.4 (0.5-3.8)	0.500
EBV DNA (≥4000 vs. <4000)	1.6 (1.0-2.5)	0.045	1.3 (1.0-1.7)	0.035	1.9 (1.2-2.9)	0.005	1.2 (0.8-1.7)	0.380
Lymph Node (CLN vs. RLN)	0.5 (0.3-0.8)	0.0015	0.5 (0.4-0.7)	<0.001	0.6 (0.4-0.8)	0.005	0.4 (0.3-0.6)	<0.001
PET/CT (Yes vs. No)	0.9 (0.7-1.0)	0.120	0.8 (0.7-0.9)	0.017	0.9 (0.7-1.1)	0.170	0.9 (0.7-1.0)	0.049
Treatment								
CCRT	Reference		Reference		Reference		Reference	
IC+CCRT	0.8 (0.5-1.3)	0.409	0.7 (0.5-0.9)	0.040	0.7 (0.4-1.1)	0.095	0.7 (0.5-1.1)	0.118
RT	1.2 (0.7-2.1)	0.573	0.9 (0.6-1.3)	0.557	0.8 (0.4-1.4)	0.370	0.6 (0.3-1.1)	0.071
IC+RT	0.4 (0.2-0.9)	0.036	0.6 (0.4-0.9)	0.013	0.7 (0.4-1.3)	0.252	0.7 (0.4-1.2)	0.154
Smoking (Yes vs. No)	1.2 (0.7-1.8)	0.530	1.2 (0.9-1.6)	0.150	1.1 (0.7-1.7)	0.710	1.2 (0.8-1.7)	0.380
Drinking (Yes vs. No)	1.2 (0.7-2.2)	0.480	1.2 (0.9-1.7)	0.280	1.3 (0.8-2.2)	0.280	1.0 (0.6-1.6)	0.960
History (Yes vs. No)	0.9 (0.4-2.0)	0.870	1.0 (0.6-1.6)	0.990	1.2 (0.6-2.3)	0.590	1.3 (0.7-2.2)	0.410

Abbreviations: CCRT, concurrent chemoradiotherapy; CI, confidence interval; CLN, cervical lymph node; DMFS, distant metastasis-free survival; EBV, Epstein Barr virus; FFS, failure-free survival; HR, hazard ratio; IC, induction chemotherapy; LDH, serum lactate dehydrogenase; LRRFS, locoregional relapse-free survival; OS, overall survival; PET/CT, [18F]-fluorodeoxyglucose positron emission tomography/computed tomography; PSM: propensity scoring matching; RLN, retropharyngeal lymph node; RT, radiotherapy.

Supplementary Table 5. Multivariable analysis of patients with EBV-DNA less than 4000 copies/ml in the PSM cohort (N=1423).

	OS		FFS		DMFS		LRRFS	
	HR (95% CI)	p	HR (95% CI)	p	HR (95% CI)	p	HR (95% CI)	p
Age (≥55 vs. <55)	3.1 (2.0-4.7)	<0.001						
PET/CT (Yes vs. No)	0.5 (0.3-0.8)	0.001	0.5 (0.4-0.7)	<0.001	0.6 (0.4-0.8)	0.004	0.5 (0.3-0.6)	<0.001
Lymph Node (CLN vs. RLN)	1.5 (0.9-2.4)	0.090	1.3 (1.0-1.7)	0.048	1.9 (1.2-2.9)	0.005		
Treatment								
CCRT			Reference					
IC+CCRT			0.7 (0.5-0.9)	0.022				
RT			0.9 (0.6-1.3)	0.641				
IC+RT			0.6 (0.4-0.9)	0.016				

Abbreviations: CCRT, concurrent chemoradiotherapy; CI, confidence interval; CLN, cervical lymph node; DMFS, distant metastasis-free survival; EBV, Epstein Barr virus; FFS, failure-free survival; HR, hazard ratio; IC, induction chemotherapy; LRRFS, locoregional relapse-free survival; OS, overall survival; PET/CT, [18F]-fluorodeoxyglucose positron emission tomography/computed tomography; PSM, propensity scoring matching; RLN, retropharyngeal lymph node; RT, radiotherapy.

Supplementary Table 6. Univariate analysis in the PSM Cohort D (N=698).

	FFS		DMFS		LRRFS		OS	
	HR (95% CI)	p	HR (95% CI)	p	HR (95% CI)	p	HR (95% CI)	p
Sex (Male vs. Female)	0.9 (0.6-1.3)	0.610	1.2 (0.6-2.2)	0.660	0.7 (0.4-1.2)	0.160	1.0 (0.5-2.1)	0.990
Age (≥55 vs. <55)	1.1 (0.7-1.7)	0.680	1.1 (0.5-2.3)	0.780	0.5 (0.2-1.2)	0.130	2.3 (1.1-4.7)	0.023
Albumin (≥40 vs. <40)	0.6 (0.3-1.3)	0.220	0.7 (0.2-2.1)	0.460	0.7 (0.2-2.3)	0.560	0.3 (0.1-0.7)	0.009
Hemoglobin (≥120 vs. <120)	2.4 (0.6-10.0)	0.230	2.6e+07 (0-Inf)	1	0.8 (0.2-3.3)	0.770	1.8 (0.2-15.0)	0.600
LDH (≥250 vs. <250)	0.6 (0.2-1.9)	0.370	0.5 (0.1-3.9)	0.530	0.5 (0.1-3.4)	0.450	1.5 (0.4-6.2)	0.590
EBV DNA (≥2000 vs. <2000)	1.3 (0.9-1.8)	0.190	1.1 (0.6-1.9)	0.840	1.3 (0.7-2.2)	0.410	1.9 (0.9-3.8)	0.058
Lymph Node （RLN vs. CLN）	0.6 (0.4-1.0)	0.067	0.3 (0.1-0.9)	0.043	0.6 (0.3-1.4)	0.220	0.8 (0.3-1.9)	0.580
rENE (Grade 3 vs. Grade 0-2)	3.2 (2.1-4.9)	<0.001	3.3 (1.7-6.3)	<0.001	3.1 (1.7-5.9)	<0.001	2.6 (1.2-5.8)	0.016
Smoking (Yes vs. No)	0.9 (0.6-1.3)	0.520	1.0 (0.5-2.0)	0.910	0.6 (0.3-1.3)	0.190	0.8 (0.4-1.8)	0.600
Drinking (Yes vs. No)	0.9 (0.6-1.5)	0.690	0.9 (0.4-2.0)	0.770	1.0 (0.5-2.1)	0.930	0.7 (0.3-1.9)	0.500
History (Yes vs. No)	1.1 (0.6-1.9)	0.830	1.1 (0.5-2.9)	0.790	1.5 (0.7-3.3)	0.350	0.3 (0.1-1.9)	0.190
Nodal Necrosis (Yes vs. No)	2.4 (1.6-3.5)	<0.001	3.5 (1.9-6.4)	<0.001	1.7 (0.9-3.2)	0.100	1.6 (0.7-3.6)	0.240
Minimal axial diameter (≥0.95 vs. <0.95)	2.9 (1.7-5.0)	<0.001	4.6 (1.6-13.0)	0.004	5.2 (1.9-14.0)	0.002	1.8 (0.8-4.1)	0.170
Maximal axial diameter (≥1.35 vs. <1.35)	2.2 (1.4-3.4)	<0.001	3.6 (1.5-8.4)	0.004	4.0 (1.7-9.4)	0.002	0.9 (0.5-1.8)	0.810
SUVmax-T (≥9.25 vs. <9.25)	1.2 (0.7-1.8)	0.520	1.3 (0.6-2.9)	0.450	0.8 (0.4-1.5)	0.520	4.5 (1.1-19.0)	0.040
SUVmax-N (≥9.35 vs. <9.35)	2.5 (1.6-3.9)	<0.001	4.6 (1.9-11)	<0.001	2.8 (1.4-5.5)	0.004	1.7 (0.8-3.6)	0.140
Treatment (IC+CCRT vs. CCRT)	0.7 (0.5-1.0)	0.075	0.6 (0.3-1.0)	0.069	0.8 (0.4-1.3)	0.340	1.4 (0.7-2.7)	0.360

Abbreviations: CI, confidence interval; CLN, cervical lymph node; CCRT, concurrent chemoradiotherapy; DMFS, distant metastasis-free survival; EBV, Epstein Barr virus; FFS, failure-free survival; HR, hazard ratio; IC, induction chemotherapy; LDH, serum lactate dehydrogenase; LRRFS, locoregional relapse-free survival; OS, overall survival; PSM, propensity scoring matching; RLN, retropharyngeal lymph node; rENE, radiologic extranodal extension; SUVmax-N, the maximal standardized uptake value of lymph node; SUVmax-T, the maximal standardized uptake value of primary tumor.

Supplementary Table 7. Multivariable analysis in the PSM Cohort D (N=698).

	FFS		DMFS		LRRFS		OS	
	HR (95% CI)	p	HR (95% CI)	p	HR (95% CI)	p	HR (95% CI)	p
rENE (Grade 3 vs. Grade 0-2)	2.4 (1.6-3.8)	<0.001	2.0 (1.0-4.0)	0.045	2.2 (1.2-4.2)	0.016		
Nodal Necrosis (Yes vs. No)	1.8 (1.2-2.7)	0.005	2.4 (1.3-4.6)	0.005				
SUVmax-N (≥9.35 vs. <9.35)	1.7 (1.0-2.8)	0.034	2.8 (1.1-7.4)	0.034				
Albumin (≥40 vs. <40)							0.4 (0.1-0.9)	0.043
SUVmax-T (≥9.25 vs. <9.25)							4.4 (1.1-18.0)	0.042

Abbreviations: CI, confidence interval; DMFS, distant metastasis-free survival; FFS, failure-free survival; HR, hazard ratio; LRRFS, locoregional relapse-free survival; OS, overall survival; PSM, propensity scoring matching; rENE, radiologic extranodal extension; SUVmax-N, the maximal standardized uptake value of lymph node; SUVmax-T, the maximal standardized uptake value of primary tumor.

Supplementary Table 8. Univariate analysis in the high-risk group of PSM Cohort D (N=454).

	FFS		DMFS		LRRFS		OS	
	HR (95% CI)	p	HR (95% CI)	p	HR (95% CI)	p	HR (95% CI)	p
Sex (Male vs. Female)	0.9 (0.6-1.4)	0.780	1.1 (0.6-2.1)	0.810	0.7 (0.4-1.4)	0.340	1.4 (0.6-3.2)	0.490
Age (≥55 vs. <55)	1.3 (0.8-2.1)	0.270	1.1 (0.5-2.3)	0.780	0.6 (0.2-1.5)	0.250	2.8 (1.3-5.9)	0.008
Albumin (≥40 vs. <40)	0.7 (0.3-1.4)	0.340	0.8 (0.2-2.5)	0.650	0.8 (0.2-2.4)	0.630	0.3 (0.1-0.8)	0.015
Hemoglobin (≥120 vs. <120)	3.8 (0.5-27.0)	0.190	2.6e+07 (0-Inf)	1.000	1.4 (0.2-10.0)	0.740	2.6e+07 (0-Inf)	1.000
LDH (≥250 vs. <250)	0.5 (0.2-1.5)	0.220	0.4 (0.1-2.9)	0.360	0.4 (0.1-2.9)	0.370	1.3 (0.3-5.5)	0.730
EBV DNA (≥2000 vs. <2000)	1.0 (0.6-1.4)	0.790	0.7 (0.4-1.3)	0.290	0.9 (0.5-1.7)	0.730	1.5 (0.7-3.2)	0.280
Lymph Node（RLN vs. CLN）	0.6 (0.3-1.3)	0.180	0.4 (0.1-1.5)	0.160	0.6 (0.2-1.8)	0.330	0.8 (0.2-2.6)	0.670
rENE (Grade 3 vs. Grade 0-2)	2.5 (1.6-3.8)	<0.001	2.1 (1.1-4.2)	0.029	2.5 (1.3-4.8)	0.007	2 (0.9-4.6)	0.093
Smoking (Yes vs. No)	0.9 (0.5-1.4)	0.520	1.1 (0.5-2.1)	0.840	0.6 (0.3-1.4)	0.220	0.9 (0.4-2.1)	0.730
Drinking (Yes vs. No)	1.1 (0.6-1.8)	0.830	0.9 (0.4-2.1)	0.770	1.3 (0.6-2.7)	0.560	0.9 (0.3-2.6)	0.840
History (Yes vs. No)	1.4 (0.7-2.6)	0.310	1.1 (0.4-3.0)	0.900	2.1 (0.9-4.7)	0.078	0.4 (0.1-2.7)	0.320
Nodal Necrosis (Yes vs. No)	1.7 (1.1-2.6)	0.0093	2.2 (1.2-4.1)	0.013	1.3 (0.7-2.4)	0.490	1.2 (0.5-2.7)	0.700
Minimal axial diameter (≥0.95 vs. <0.95)	2.1 (0.9-4.7)	0.088	1.6 (0.5-5.1)	0.440	8e+07 (0-Inf)	1.000	0.8 (0.3-2.3)	0.700
Maximal axial diameter (≥1.35 vs. <1.35)	1.3 (0.7-2.3)	0.390	1.5 (0.6-3.8)	0.410	4.1 (0.9-17.0)	0.051	0.5 (0.2-1.1)	0.075
SUVmax-T (≥9.25 vs. <9.25)	1.0 (0.6-1.7)	0.970	1.2 (0.5-2.7)	0.670	0.7 (0.3-1.4)	0.300	2.9 (0.7-12.0)	0.140
SUVmax-N (≥9.35 vs. <9.35)	1.2 (0.5-2.7)	0.710	1.0 (0.3-3.2)	0.980	3.3 (0.5-24.0)	0.2400	0.5 (0.2-1.7)	0.290
Treatment (IC+CCRT vs. CCRT)	0.6 (0.4-0.9)	0.007	0.5 (0.3-0.9)	0.023	0.5 (0.3-0.9)	0.038	1.4 (0.6-2.9)	0.430

Abbreviations: CI, confidence interval; CLN, cervical lymph node; CCRT, concurrent chemoradiotherapy; DMFS, distant metastasis-free survival; EBV, Epstein Barr virus; FFS, failure-free survival; HR, hazard ratio; IC, induction chemotherapy; LDH, serum lactate dehydrogenase; LRRFS, locoregional relapse-free survival; OS, overall survival; PSM, propensity scoring matching; RLN, retropharyngeal lymph node; rENE, radiologic extranodal extension; SUVmax-N, the maximal standardized uptake value of lymph node; SUVmax-T, the maximal standardized uptake value of primary tumor.

Supplementary Table 9. Multivariable analysis in the high-risk group of PSM Cohort D (N=454).

	FFS		DMFS		LRRFS		OS	
	HR (95% CI)	p	HR (95% CI)	p	HR (95% CI)	p	HR (95% CI)	p
rENE (Grade 3 vs. Grade 0-2）	2.5 (1.6-3.9)	<0.001	2.1 (1.1-4.1)	0.034	2.6 (1.4-5.1)	0.004		
Nodal Necrosis (Yes vs. No)	1.7 (1.1-2.5)	0.015	2.1 (1.1-3.9)	0.018				
Treatment (IC+CCRT vs. CCRT)	0.5 (0.4-0.8)	0.003	0.5 (0.2-0.9)	0.015	0.5 (0.3-0.9)	0.025		
Age (≥55 vs. <55)							2.6 (1.2-5.5)	0.015
Albumin (≥40 vs. <40)							0.3 (0.1-0.9)	0.032

Abbreviations: CI, confidence interval; DMFS, distant metastasis-free survival; FFS, failure-free survival; HR, hazard ratio; LRRFS, locoregional relapse-free survival; OS, overall survival; PSM, propensity scoring matching; rENE, radiologic extranodal extension; SUVmax-N, the maximal standardized uptake value of lymph node; SUVmax-T, the maximal standardized uptake value of primary tumor.
